# Infectious keratoconjunctivitis in wild Caprinae: merging field observations and molecular analyses sheds light on factors shaping outbreak dynamics

**DOI:** 10.1186/s12917-017-0972-0

**Published:** 2017-03-04

**Authors:** Giuseppina Gelormini, Dominique Gauthier, Edy M. Vilei, Jean-Paul Crampe, Joachim Frey, Marie-Pierre Ryser-Degiorgis

**Affiliations:** 1 Centre for Fish and Wildlife Health (FIWI), Vetsuisse Faculty, University of Bern, Bern, Switzerland; 2 Institute of Veterinary Bacteriology, Vetsuisse Faculty, University of Bern, Bern, Switzerland; 3 Laboratoire Vétérinaire des Hautes Alpes, Gap, France; 4 Parc National des Pyrénées, Tarbes, France

**Keywords:** Molecular epidemiology, *Mycoplasma conjunctivae*, Persistence, Resurgence, Ocular disease, Disease spread, Chamois, Ibex

## Abstract

**Background:**

Infectious keratoconjunctivitis (IKC) is an ocular infectious disease caused by *Mycoplasma conjunctivae* which affects small domestic and wild mountain ruminants. Domestic sheep maintain the pathogen but the detection of healthy carriers in wildlife has raised the question as to whether *M. conjunctivae* may also persist in the wild. Furthermore, the factors shaping the dynamics of IKC outbreaks in wildlife have remained largely unknown. The aims of this study were (1) to verify the etiological role of *M. conjunctivae* in IKC outbreaks recorded between 2002 and 2010 at four study sites in different regions of France (Pyrenees and Alps, samples from 159 Alpine ibex *Capra ibex*, Alpine chamois *Rupicapra rupicapra* and Pyrenean chamois *Rupicapra pyrenaica*); (2) to establish whether there existed any epidemiological links between the different regions through a cluster analysis of the detected strains (from 80 out of the 159 animals tested); (3) to explore selected pathogen, host and environmental factors potentially influencing the dynamics of IKC in wildlife, by joining results obtained by molecular analyses and by field observations (16,609 animal observations). All of the samples were tested for *M. conjunctivae* by qPCR, and cluster analysis was based on a highly variable part of the *lppS* gene.

**Results:**

We documented infections with *M. conjunctivae* in epidemic and endemic situations, both in symptomatic and asymptomatic animals. The identified *M. conjunctivae* strains were site-specific and persisted in the local wild population for at least 6 years. In epidemic situations, peaks of cases and disease resurgence were associated with the emergence of new similar strains in a given area. Social interactions, seasonal movements and the landscape structure such as natural and anthropogenic barriers influenced the spatio-temporal spread of IKC. Adults were more affected than young animals and host susceptibility differed depending on the involved strain.

**Conclusion:**

Our study indicates that IKC is a multifactorial disease and that *M. conjunctivae* can persist in wildlife populations. The disease course in individual animals and populations is influenced by both host and mycoplasma characteristics, and the disease spread within and among populations is shaped by host behavior and landscape structure.

**Electronic supplementary material:**

The online version of this article (doi:10.1186/s12917-017-0972-0) contains supplementary material, which is available to authorized users.

## Background

Infectious keratoconjunctivitis (IKC) in Caprinae is a common disease characterized by varying ocular signs ranging from mild conjunctivitis with discrete lachrymation to severe keratitis with cornea perforation, resulting in irreversible blindness, associated behavioral changes and, eventually, death [1–5]. It affects both wild and domestic Caprinae but severe signs are more frequent in wildlife [6]. In wild ruminants, the disease has been known for nearly a century and reported to occur in at least seven European countries (Switzerland, Italy, France, Austria, Slovenia [7], Spain [8], Norway [9]), in North America [10, 11] and in Oceania (New Zealand [12], Australia [13]).

IKC has been reported to be the cause of potentially important economic losses for farmers because of treatment costs, weight loss and mortality due to falls or drowning in affected animals [14]. In wildlife, the disease can result in high mortality, reaching up to 30% of the estimated population size [2, 15], which is perceived as problematic due to the local significance of wild ungulates as game or as cultural symbol, for ecotourism and species conservation. In the Alps in particular, this disease has an emblematic character as it frequently occurs, is easily recognized by observers and is perceived to be a cause of suffering in animals, in particular when the cornea is perforated, animals are disoriented and numerous carcasses are found. There is therefore a strong interest among wildlife managers and farmers in collecting more knowledge on IKC dynamics in domestic and wild animal populations.

*Mycoplasma conjunctivae* has been isolated from domestic and wild animals with IKC signs in various parts of the world, including Europe [16], North America [10, 11], Africa [17], Asia [18] and Oceania [19, 20], and IKC was successfully induced by inoculation of *M. conjunctivae* under experimental conditions [3, 21]. Most recently, the existence of a strong association between *M. conjunctivae* infection and IKC signs has also been documented in epidemiological surveys [4, 22] and *M. conjunctivae* is now recognized as the major etiological agent of IKC in Caprinae species.

Transmission of *M. conjunctivae* occurs by direct contact [23] and possibly by eye-frequenting insects [24, 25], with subsequent rapid spread within a herd [1]. Interspecific transmission of *M. conjunctivae* has been documented both experimentally and under field conditions [3, 26]. The disease is endemic in populations of small domestic ruminants, and healthy carriers have been implicated in IKC introduction into sheep herds [27, 28]. Sheep have therefore been proposed as reservoir for *M. conjunctivae* and as the main source of infection for wild ungulates on summer pastures, while wild ungulates have been considered to be spill-over hosts, i.e., as hosts susceptible to infection but unable to maintain the pathogen on their own [3, 6, 26, 29]. However, the presence of healthy carriers has recently been documented in free-ranging Alpine ibex (*Capra ibex*) and chamois (*Rupicapra spp.*) populations, both in the Alps and the Pyrenees [4, 22, 30] raising the question as to whether *M. conjunctivae* may also persist in wildlife [4, 15]. Furthermore, it has been discussed that IKC may be a multifactorial disease, with host, pathogen and environmental factors playing a role in disease occurrence and severity [4, 31].

In France, the first IKC cases were documented in 1935 and 1942 in the Ecrins National Park and in 1974 in the Vanoise National Park. The first important outbreak was documented in 1977 in the Bauge National Hunting Reserve, and in 1981 in the Pyrenees National Park [32]. Several IKC outbreaks have been described [15, 29, 33] and some authors have proposed that the disease is endemic in wild populations, occasionally turning into epidemics with varying levels of mortality [15, 32]. However, so far *M. conjunctivae* has not been reported from affected chamois or ibex in that country. Furthermore, it has often been unclear whether an epidemiological link existed among affected subpopulations and by which mechanisms IKC spread occurred. Overall, the role of sheep in IKC re-emergence in wildlife remains controversial, and the potential role of intraspecific social interactions, landscape structure and varying virulence of *M. conjunctivae* strains in the dynamics of IKC outbreaks in wildlife has been poorly investigated.

The aims of this study were (1) to verify the implication of *M. conjunctivae* in IKC outbreaks in wild ruminants in France, (2) to establish whether there were epidemiological links between the different outbreaks that occurred in France over a period of 9 years, and (3) to explore selected pathogen, host and environmental factors potentially influencing the dynamics of IKC in wildlife. We hypothesized that *M. conjunctivae* strain characteristics, intraspecific social interactions and landscape structure may all influence the dynamics of IKC in wild populations. We analyzed documented field observations of IKC events in France between 2002 and 2010 and carried out molecular investigations on animal samples collected in the affected areas during the same time period.

## Methods

### Study areas

**Fig. 1:**
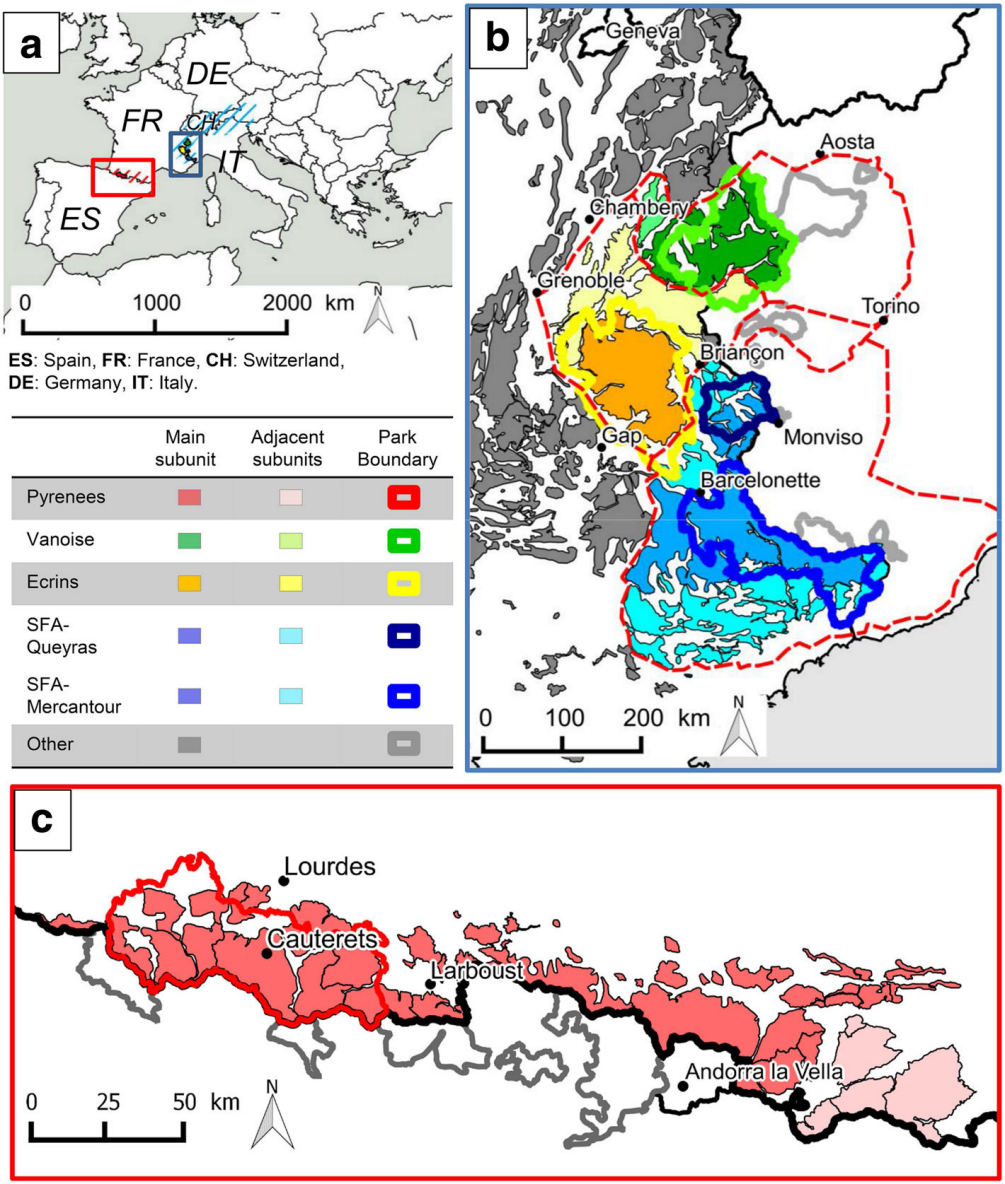
Overview of the study areas. **a** Map of west-central Europe depicting the location of the mountain ranges of the Pyrenees (*red* striped area) and of the Alps (*blue* striped area) and the corresponding regions including the study areas (*red* and *blue rectangles*, respectively). *Light grey* surfaces represent the Atlantic Ocean and seas. **b** and **c** Close-ups of these regions, illustrating the distribution of wild Caprinae in France (filled areas). *Solid thick black lines* are national boundaries. *Dashed red lines* delimit epidemiological units in the Alps as defined in the text. In the Pyrenees, the entire mountain chain is considered to be a single epidemiological unit. *Solid colored* and *grey lines* correspond to the boundaries of national and regional natural parks relevant for the study. Different colors of surfaces and solid lines correspond to different epidemiological subunits and parks, respectively, on the French side of the national boundary. Shades of the same color correspond to a same study area (*Green*: Vanoise; *Yellow*: Ecrins; *Blue*: Southern French Alps, including the parks of Queyras and Mercantour; *Red*: Pyrenees). Shades of dark grey correspond to parks and subunits outside the study areas. *Black dots* and names indicate main cities

The study was conducted in four areas in France. These study areas corresponded to distinct mountain regions located in the Pyrenees (one area) and in the Alps (three areas: Vanoise, Ecrins and Southern French Alps; Fig. 1). We tentatively defined epidemiological units considering geographical barriers, spatial occupation by wild Caprinae and ecological corridors between population nuclei according to data collected between 2005 and 2010 by the French National Office for Hunting and Wildlife (Office National de la Chasse et de la Faune Sauvage, ONCFS; Additional file 1). Wild Caprinae species included Pyrenean chamois (*Rupicapra p. pyrenaica*) and mouflons (*Ovis gmelini musimon*) in the Pyrenees, and Alpine chamois (*Rupicapra r. rupicapra*), Alpine ibex (*Capra i. ibex*) and mouflons in the Alps. Spatial distribution and estimated population densities of the four species are given in Additional file 1. Accordingly, there was only one epidemiological unit in the Pyrenees whereas there were several in the Alps. Each epidemiological unit was further divided into subunits of various sizes, with a main (largest) subunit and 2–14 adjacent subunits. Contacts were considered to be impossible between wild Caprinae from the Pyrenees and the Alps, unlikely but not impossible among wild Caprinae from different epidemiological units within the Alps, and likely but not documented between Caprinae from different subunits within a same epidemiological unit.

**Fig. 2:**
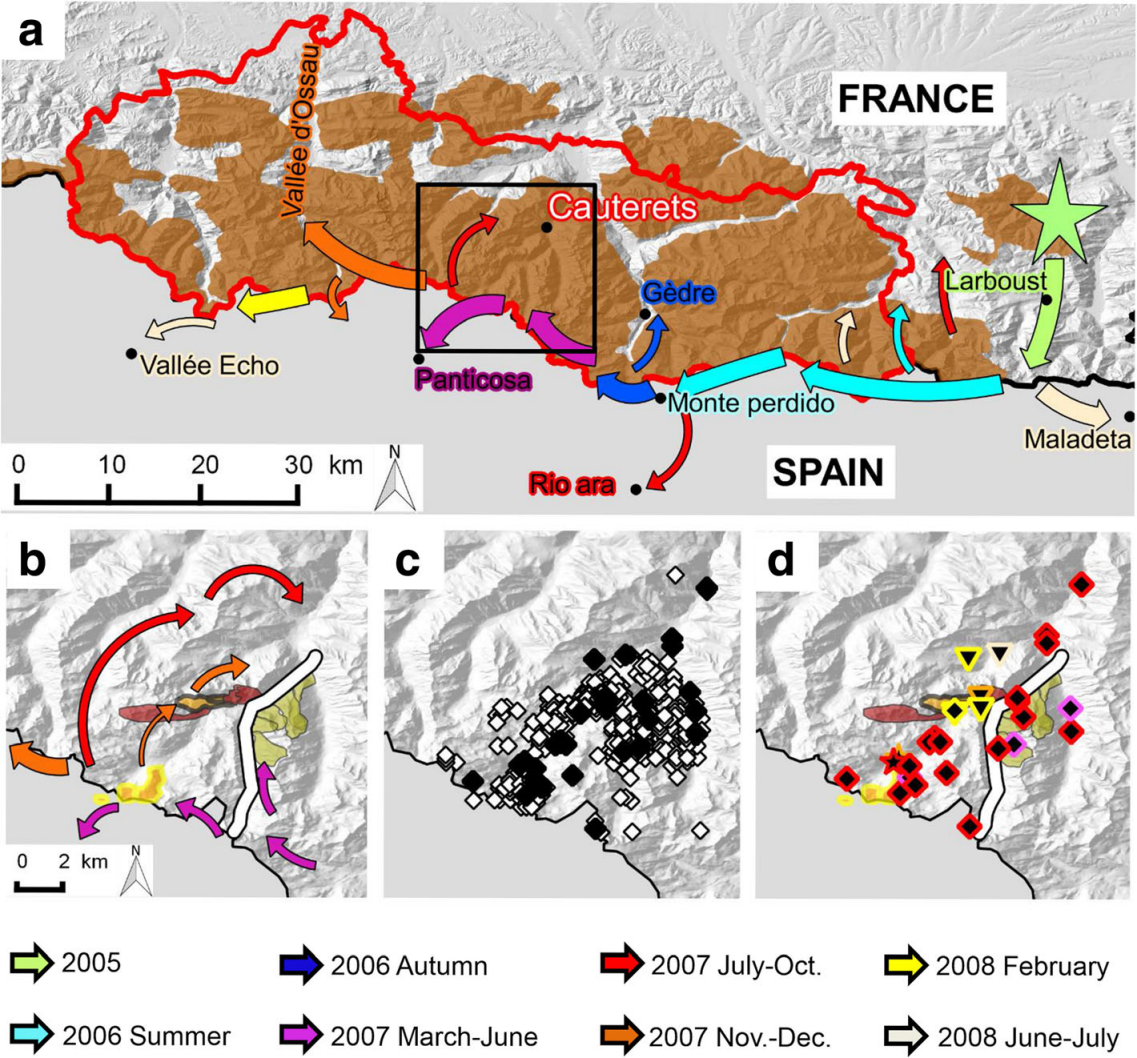
Outbreak of infectious keratoconjunctivitis (IKC) in the French Pyrenees. **a** Distribution of Pyrenean chamois (*brown* surfaces) in the Pyrenees National Park (PNP, within the solid *red line*) and adjacent areas, and spatio-temporal progression of the IKC outbreak in 2005–2007 (*colored arrows*). Colors correspond to defined time periods. The outbreak started in 2005 in Larboust (*green star*), subsequently followed the mountain range along the border between France and Spain, and spread into the perpendicular French and Spanish valleys. The epidemic front reached the PNP in the summer of 2006 and the sectors of Cauterets (*black square*) in the summer of 2007 and came to a stop in Spain in 2008. **b–d** Close-ups of the two zones of Cauterets, which are separated by the Gaube valley (*thick white line*). **b** Spatio-temporal spread of the outbreak (*arrows*). Colored surfaces represent the areas occupied by the different groups of female Pyrenean chamois. The eastern zone was affected first (*purple arrows*, *yellow* surfaces) but the outbreak simultaneously progressed along the mountain range and reached the herds of the western zone grazing near the boundary (*purple arrows*, *orange* surfaces with *yellow* border). These herds brought the disease to their winter pastures in the western zone (thin *orange arrows*, *orange* surfaces with *black* border), transmitting the disease to other groups in this area (*dark red* surfaces). **c** *White* diamonds represent the observed chamois affected by IKC although not sampled. *Black diamonds* are chamois tested for *Mycoplasma conjunctivae* by PCR. **d** Geographical origin of the sequenced positive samples and location of the chamois herds (colored surfaces, see also **b**) west and east from the Gaube valley. The three detected strains are represented by: Diamonds = PYR_07-08a; Stars = PYR_07b; Reversed triangles = PYR_07-08c. The colored border around these symbols corresponds to different time periods based on the same color code as for the *arrows* in **a** and **b**

The Pyrenees (Figs. 1 and 2; 42° 18′ N to 43° 06′ N and 1° 03′ W to 2° 42′ E) are a mountain range shared between France and Spain. Topologically, the mountains are like a kind of fish bone with the border between France and Spain representing the “spinal cord” where contacts between the different subpopulations of Pyrenean chamois are only possible via this “spinal cord” [34]. The Pyrenees National Park (PNP, 252′100 ha; hunting prohibited in the core area, 47′707 ha) is located on the French side (Figs. 1c, and 2a). For this study, we took into consideration all available information on IKC outbreaks in the French Pyrenees, but most samples were collected in the core area of the PNP, in the western and eastern zones of Cauterets, which are separated by the Gaube valley (Fig. 2b). In Cauterets, ecological studies on Pyrenean chamois have been carried out for about 20 years. The social structure and landscape use of this portion of the population has been well-documented [34–36] and nearly 180 chamois were marked at the time of the IKC epidemic.

**Fig. 3:**
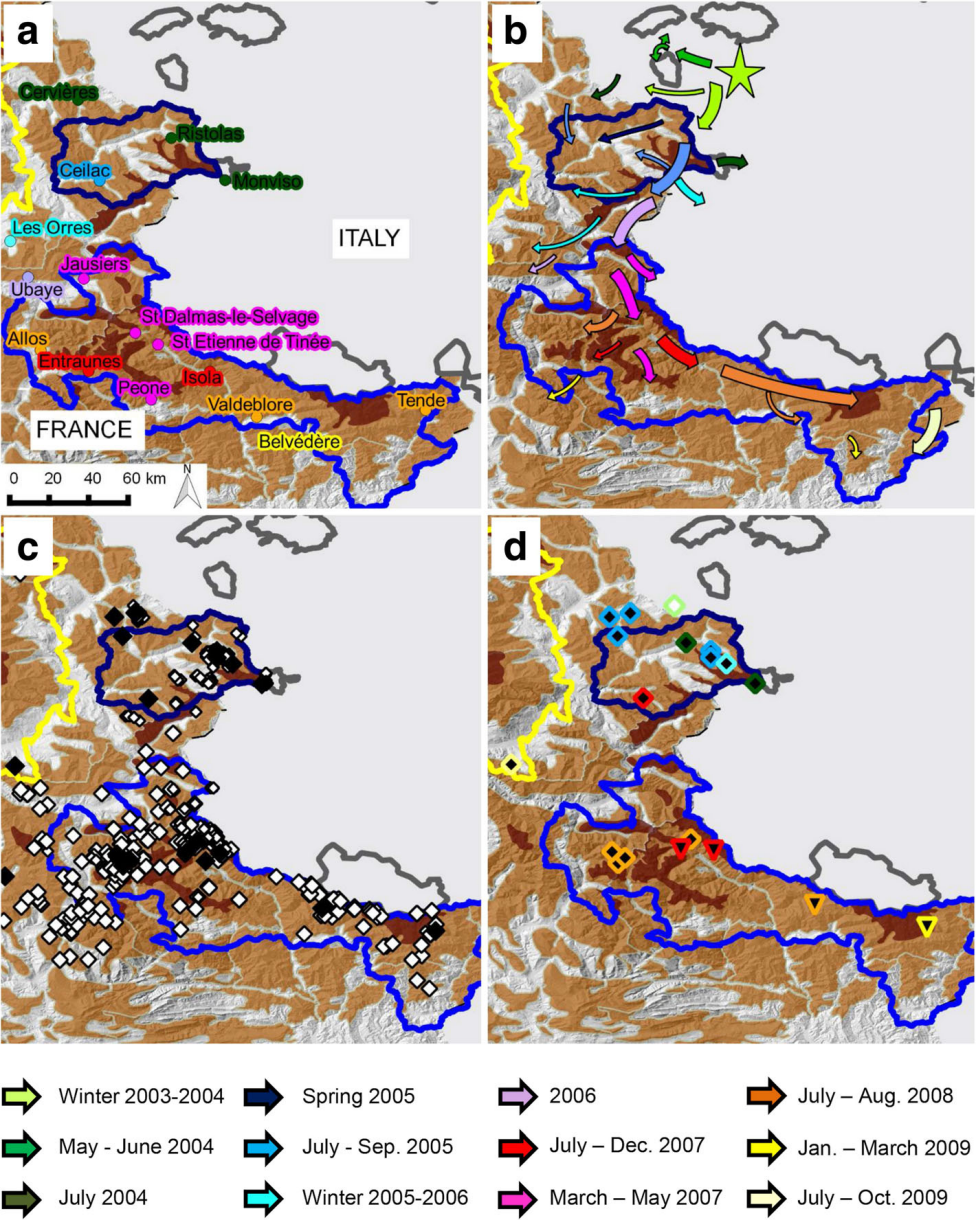
Outbreak of infectious keratoconjunctivitis (IKC) in the Southern French Alps. *Brown* surfaces illustrate the distribution of wild Caprinae (*light brown*: chamois; *dark brown*: ibex) in the Queyras National Park (QNP, *dark blue solid line*), Mercantour National Park (MNP, royal *blue solid line*) and adjacent areas in France. The *yellow solid line* corresponds to the south-eastern border of the Ecrins National Park. **a** and **b** Spatio-temporal progression of the IKC outbreak in 2003–2009 (*colored dots*, names and *arrows*). Dots and names indicate the location of important places. *Arrows* depict the spatial progression of the epidemic front. Each color corresponds to a defined time period. The outbreak started in 2003 in Italy (*green star*) and spread to the south. In 2005 the entire QNP was affected. In 2006 only a few cases were recorded. In 2008–2009 the entire MNP was affected. **c** *White* diamonds represent the observed chamois and ibex affected by IKC although not sampled, whereas *black diamonds* are animals tested for *Mycoplasma conjunctivae* by PCR. **d** Geographical origin of the sequenced positive samples. The two isolated strains are represented by different symbols: Diamonds = MER_04-09; Reversed triangles = MER_07-09. The colored borders around these symbols correspond to different time periods based on the same color code as for the arrows and localities in **a** and **b**. The Italian strain (n°84 [70], *white* diamond with *green border*) is identical to one of the two French strains (*black* diamonds and reverse triangles)

The study area “Southern French Alps” (Figs. 1 and 3; 43° 38′ N to 44° 56′ N and 6° 12′ E to 7° 42′ E) is part of the mountain range shared by France and Italy. It comprises two main mountain massifs: The Queyras (including the Queyras Regional Natural Park, 58,000 ha, and the two regions of Cervières and Ristolas) in the north, and the Mercantour (including the Mercantour National Park, 210,000 ha, and surrounding regions) in the south. Common law Hunting is practiced everywhere except for the hunting reserve of Ristolas (2295 ha) and the core area of the Mercantour National park (68,500 ha, hunting prohibited). In the reserve of Ristolas, studies on population dynamics have been conducted but without marking procedures. In the Mercantour National Park, numerous ungulates have been marked for ecological studies, including 260 chamois and 31 mouflons.

The study area “Ecrins” (Fig. 1b; 44° 33' N to 45° 28′ N and 5° 45′ E to 6° 58′ E) corresponds largely to the Ecrins National Park (272’000 ha). The area comprises a central mountain massif, with long and deep valleys characterized by large areas used as livestock pasture converging to the heart of the massif, made of rocky slopes and glaciers. Hunting is practiced everywhere except in the core area of the park (91,800 ha). The study area “Vanoise” (Fig. 1; 45° 12′ N to 45° 43′ N and 6° 19′ E to 7° 12′ E) corresponds to the Vanoise mountain massif, including the Vanoise National Park (200,000 ha, hunting prohibited in the core area, 52,900 ha) and the Encombres massif. The region is located in the heart of the northern Alps and is adjacent to the Italian National Park Gran Paradiso. In the Vanoise National Park, ibex have been caught and marked since 1981, with around 300 animals marked within the region where animals were sampled for this study (including 165 within the past 10 years).

Domestic sheep are found in all four study areas. Some live in the parks all year round; others are brought from different French departments (Additional file 2) each summer from June to October (transhumance) to graze on alpine pastures.

IKC epidemic events in wildlife have previously been reported in the Pyrenees, the Southern French Alps and the Vanoise [8, 15, 29] but never in the Ecrins, where to date only sporadic cases have occurred.

### Field observations

**Fig. 4:**
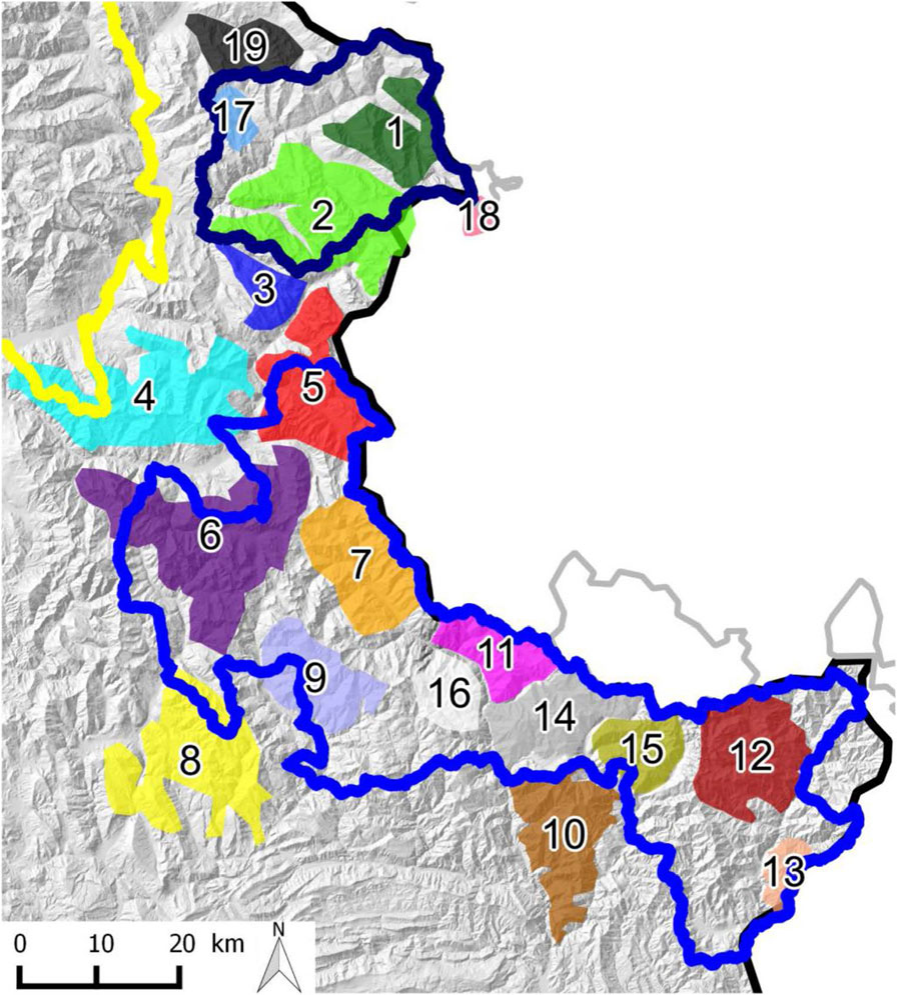
Observation sectors in the Southern French Alps. Map depicting the 19 sectors (*colored areas*) defined to perform field observations and the two national parks (Queyras, *dark blue solid line*; Mercantour, royal *blue solid line*) comprised in the study area Southern French Alps

In all areas, information on the local IKC situation was collected within the framework of general surveillance, including reports by the public, hunters and gamekeepers on observations of live and dead animals with eye lesions or other clinical signs. In addition, targeted surveillance schemes were implemented once an increase of IKC cases was registered but this was done only in sectors with professional game keepers able to carry out the work. Consequently, detailed data were provided only for small zones or sectors of the two study areas where severe IKC epidemics have occurred: the Pyrenees (two zones of Cauterets, Fig. 2) and the Southern French Alps (19 sectors, Fig. 4). Sectors were defined according to the designed itineraries (see below), to chamois, mouflons and ibex distribution (ONCFS, Additional file 1) and to IKC waves.

Targeted observations were performed on foot, using field glasses and telescopes. Observation protocols were standardized within each region and included fields to record at least the date of observation, geographical location of the observed animals and their species, age and sex. Observers were additionally asked to describe ocular signs for all observed IKC cases. Sex was determined based on morphological traits, i.e., body size and morphology, horn size and shape, penis and social behavior [37]. Age was estimated based on horn length, hair color and behavior. Recorded eye lesions included ocular discharge, corneal opacity, perforation and neovascularization. Orphans were identified based on 1) behavioural differences compared to kids following their mother, 2) the number of kids and females within a social group, and 3) the known reproductive status of marked females.

In the Pyrenees, observations were carried out in the two zones of Cauterets during the entire period of the IKC outbreak that began in March 2007 and ended in November 2008. Observations were performed on given itineraries at varying times of day, approximately every 3 days, with a decrease in frequency as the epidemic wave vanished, i.e., when new cases were not observed any more. The area in the north-west of the Gaube valley of Cauterets was observed with particular intensity because it corresponded to the study area of other long-term investigations (e.g. [34–36]). All observations were recorded with the same data sheet by few well-trained gamekeepers.

In the Southern French Alps, itineraries were designed with the goal to observe at least 100 animals (Alpine ibex, Alpine chamois, mouflon) per observation day and per mountain massif (Queyras and Mercantour). This number of 100 was arbitrarily set to estimate the prevalence of symptomatic animals. Each itinerary was covered at dawn, with a frequency of observation varying from daily to every 2 weeks, the highest observation frequency corresponding to the outbreak peaks. Observations were carried out by a range of observers (hunters, gamekeepers, veterinarians, biologists and other trained people). Additionally, we considered data compiled in Italy in the region adjacent to our study area to describe the progression of the outbreak which spread over that region [38].

Overall, 4077 animal-observations were recorded in the Pyrenees, and 11,861 in the Southern French Alps, excluding mouflons because of the very low number of observations for this species.

### Sampling and animals

Samples were collected in all four study areas. Sampling was carried out by gamekeepers, hunters, veterinarians or biologists on 159 live and dead free-ranging Pyrenean chamois, Alpine chamois and Alpine ibex with and without IKC signs (Table 1). No animal was killed for the purpose of the study. Samples consisted of 19 heads, 96 eyes (from 63 dead animals) and 94 conjunctival swabs (from 77 dead or live animals) that were kept frozen at −20 °C until analysis. Date of sampling, location, species, age, sex and ocular signs (see below) were recorded for each sampled animal. Age was estimated based on horn ring counts as well as on tooth wear for animals less than 4 years old [37, 39].

**Table 1:** Sampled animals and presence of signs of infectious keratoconjunctivitis (IKC)

	Pyrenees		Vanoise		Ecrins		Southern French Alps			Total
	IKC signs	No IKC signs	IKC signs	No IKC signs	IKC signs	No IKC signs	IKC signs	No IKC signs	No data	
Alpine chamois	-	-	-	-	8	3	26	7	10	54
Pyrenean chamois	38	-	-	-	-	-	-	-	-	38
Ibex	-	-	13	45	-	-	2	-	-	60
Total	38	-	13	45	8	3	32	10	10	159
Total per study area	38		58		11		53			

Numbers of sampled animals are given per species and study area (Pyrenees, Vanoise, Ecrins, and Southern French Alps).

### Laboratory analyses

All frozen swabs, eyes, and heads were thawed, and swabs were taken from the organic material (98 swabs from defrosted eyes and 36 swabs from eyes of defrosted heads). Subsequently, DNA was extracted from the swabs using the method described by Vilei et al. [40] (*n* = 210). For swabs collected after October 2010 (*n* = 18) DNA was extracted with the Guanidium method [41], which includes a DNA-purification step after the DNA extraction, in contrast to the former method.

Subsequently, samples were tested for *M. conjunctivae* by qPCR according to previously established protocols [40]. Mycoplasmal DNA of all samples positive for *M. conjunctivae* was then amplified by nested PCR according to the method described by Belloy et al. [26], except that different primers were used (listed in Table 2) because a mutation of 1 bp was observed in some French strains at the binding site of the primers used by Belloy et al. Samples from which DNA extraction was performed with the method of Vilei et al. [40] and for which we could not obtain a valid result by qPCR (positive or negative) because they contained inhibitory substances, such as blood or hairs, were diluted at 1/10 and 1/100 with pyogen-free water. This procedure delivered a final valid result for each animal. An increase of the MgCl_2_ concentration of the PCR’s premix to 3 mM also resulted in better amplifications. All PCR products were purified with the High Pure PCR Product Purification Kit (Roche Diagnostics, Rotkreuz, Switzerland) for subsequent DNA sequence analysis.

**Table 2:** Primers used for this study

Primer	Sequence (5′-3′)	Position^a^	Use	Reference
Serstart3	TTTAGTAGACTCCACTTCACC	3731–3751	PCR	Belloy et al. [26]
Serstart2	CACTATACTTAACAGATAGTCC	3781–3802	Nested PCR, Sequencing	Mavrot et al. [4]
Serstart0	ATACTCAAAGTGGAAATAATGGAA	3979–4002	Sequencing	This study
Serend0	GCAACAACAATAGTAAGAGCAG	4743–4764	Sequencing	This study
lppTA2	TTTGATCTCTCCACCTTCAGC	5113–5093	PCR	Mavrot et al. [4]
lppTA	GGCACTAATAGTGCGTAATTC	5065–5045	Nested PCR	Mavrot et al. [4]

^a^Position with reference to nucleotide sequence of lppS and lppT of *M. conjunctivae* HRC/581 T (GenBank accession number AJ318939)

DNA sequence determination was performed using the BigDye termination cycle sequencing kit (Applied Biosystems, Forster City, CA, USA) with the sequencing primers Ser_start2, Ser_start0 and Ser_end0 (Table 2). The sequence analyzed is a highly variable region of the gene *lppS*, which is situated between positions 4013 and 4663 with reference to nucleotide sequence of *lppS* and *lppT* of *M. conjunctivae* type strain HRC/581^T^ (GenBank accession number AJ318939). Belloy et al. [42] demonstrated that the use of this domain coding for serine repeats of *lppS* is a valuable approach for molecular epidemiology. To be able to compare the obtained fragments of this study with those from former investigations, we used the same method. In the present study, the term of “strain” refers to this specific sequence. All but six of the positive samples could be sequenced (*n* = 81). Sequencing products were analyzed on an ABI Prism 3100 genetic analyzer (Applied Biosystems) and edited using the DNA sequence analysis software Sequencher (GeneCodes, Ann Arbor, MI, USA).

Cluster relationships between strains were assessed first by alignment with the MAFFT-program (http://mafft.cbrc.jp/alignment/server) and then different phylogenetic programs were used from the websites http://mobyle.pasteur.fr and www.phylogeny.fr. The cluster analyses were compared with field observations and sequencing results in order to find the best associations between the strains. The final tree was represented with the program Bionumeric 6.6 (Applied Maths, Kortijk, Belgium) and cophenetic correlation was assessed to estimate the branch quality. Except for those identified in the present study, the strains used for the cluster analysis are a selection (one per cluster, all compared by using the method described above) of the 200 strains identified at the Institute of Veterinary Bacteriology of Bern since 1994, including the type strain HRC/581^T^ and 31 strains from Europe, i.e. from Austria (*n* = 3 [3, 26]), Switzerland (*n* = 16 [4, 26]), Italy (*n* = 6 [26]), Croatia (*n* = 2 [3]), and Spain (*n* = 4 [8]). Only strains with low divergence (>87%), which were constant from one algorithm to another, were represented in the definitive tree as well as strains from Spain which were sampled at the same period but in another region of the Spanish Pyrenees. The reference strain HRC/581^T^ was added for comparison among the different clusters.

### Data analysis

Four age/sex categories were distinguished (kids: first year of life; yearling: second year of life; adult males and adult females: 2 years and older). Ocular signs were classified into four categories according to Mavrot et al. [4]: (0) asymptomatic (no noticeable ocular change), (1) mild signs (subtle to marked ocular discharge in the absence of visible corneal lesions), (2) moderate signs (ocular discharge and corneal opacity) and (3) severe signs (ocular discharge and corneal lesions including neovascularization up to perforation).

Maps were drawn with the program quantum GIS (QGIS) version 2.4.0 [43]. Maps showing the distribution of wild ungulates are based on documents from the Office national de la chasse et de la faune sauvage (ONCFS) [44]. Maps of France were provided by the Institut national de l’Information géographique et forestière (IGN) [45]. Distances were calculated drawing lines as the crow flies. Surfaces indicated as affected by an IKC outbreak refer to the surface of the affected epidemiological units.

Data handling was done in MS Excel^©^ spread sheets. Prevalences were calculated assuming test sensitivity and specificity of 100%. Monthly prevalence was only estimated for selected sectors (one sector in the Pyrenees and 19 sectors in the Mercantour, Fig. 4) and time periods with high observation pressure, i.e. when at least 50 ibex or chamois per month had been observed. The two-tailed Fisher’s exact test (FET) was used to determine differences in prevalence of infection among age/sex categories, study areas and time periods, as well as differences in diagnostic success obtained with different sample materials or DNA extraction techniques. Level of significance was set at *p* < 0.05.

## Results

### Presence of M. conjunctivae

Animals positive to *M. conjunctivae* were found in all study areas. At individual level, 72 out of 87 (82.8%) symptomatic chamois and ibex were positive, including 13 ibex from the Vanoise and six chamois from the Ecrins. Furthermore, four out of 37 asymptomatic ibex captured from two adjacent population nuclei in the Vanoise (Modane and Champagny) were positive (two from each subpopulation). These animals were marked 2 years after the IKC epidemic and followed up for several years within the framework of another study [46]; they were never observed with IKC signs. The other eight asymptomatic individuals tested in this study were negative for *M. conjunctivae*.

**Fig. 5:**
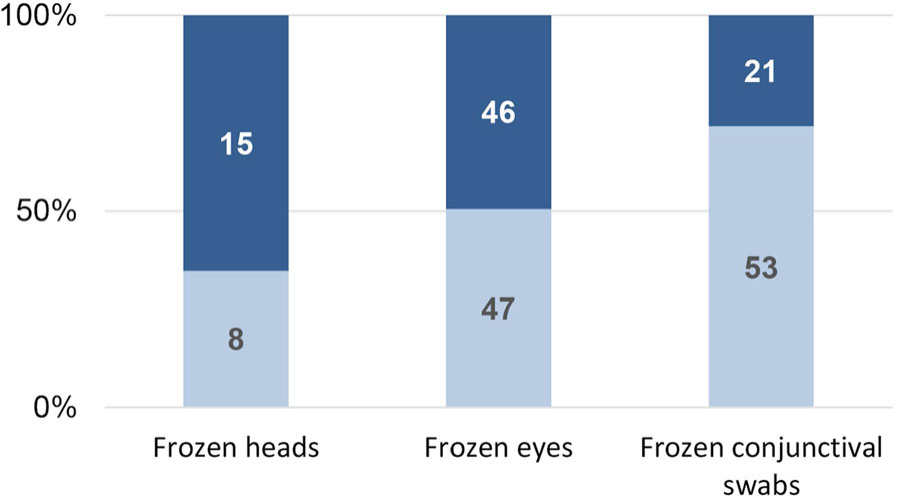
Testing success by quantitative PCR, for different sample materials. qPCR was performed after DNA extraction according to Vilei et al. [40]. The *light blue* portion of the bars corresponds to the percentage of valid results (negative or positive) obtained for the given material. The *dark blue* portion represents the percentage of undetermined results (mainly due to inhibition). Numbers indicated in the bars give the total of analysed samples for each portion

From a technical point of view, our data pointed at significant differences in the quality of the results obtained, namely the number of positive/negative vs. non-interpretable results, depending both on the type of samples and on the extraction method. Considering only the results generated with the method of Vilei et al. [40], the percentage of interpretable results obtained in the first run was significantly higher with swabs taken on fresh material and kept frozen until analysis (71.6%) than with swabs collected from thawed eyes (50.5%, *P* = 0.0069) or heads (34.8%, *P* = 0.0025) (Table 1, Fig. 5). Regarding the DNA extraction protocol, the chances of obtaining an interpretable result in the first run and a trustful Ct-value in the qPCR were higher with the method of Bürki et al. [41] (100%; 18/18 swabs including six swabs taken on frozen eyes, and 30/30 swabs from fresh tissues not included in this study; G. Gelormini, unpubl. data) than with the method previously established by Vilei et al. [40] (51.4%, 108/210 swabs; *P* = 0.0000).

### Disease spread and cluster analysis

**Fig. 6:**
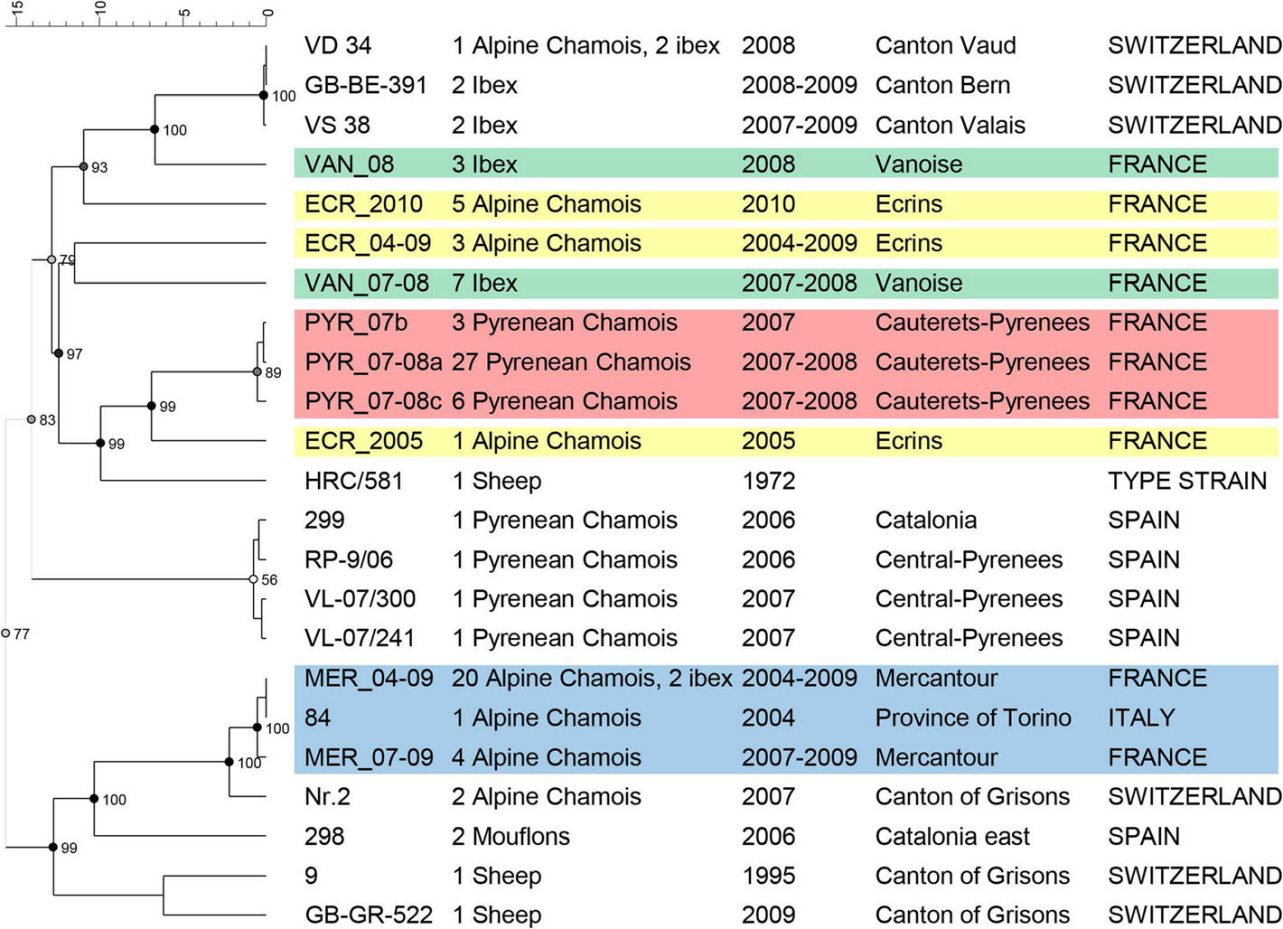
Cluster analysis of *Mycoplasma conjunctivae* strains detected in France in 2004–2010. The 10 identified French strains were compared with 13 strains selected out of 200 from the databank of the Institute of Veterinary Bacteriology at the University of Bern ([14, 34, 71], unpublished strains) (each strain staying for one cluster). Colors correspond to the geographical origin of the samples in which the listed strains were identified. *Green*: Vanoise; *Yellow*: Ecrins; *Blue*: Southern French Alps

The epidemic situation strongly differed among study areas, and sequencing revealed separate strain clusters for each study area (Fig. 6). GenBank accession numbers and metadata of the strain sequences are given in Additional file 3.

#### Pyrenees

The IKC outbreak in Pyrenean chamois lasted 4 years. It began in 2005 in Larboust (Fig. 2a), reached the France-Spain border in 2006 (12 km as the crow flies), followed the main mountain range and spread both in Spain and France through the perpendicular valleys. It covered 46 km as the crow flies in 2006 and an additional 30 km in 2007. Cases were regularly observed during the 4 years. Disease signs were typical, i.e., ocular discharge with or without corneal opacity, perforation and/or neovascularization [4], and often severe (blind animals with perforated cornea) during the entire outbreak, with marked associated mortality [34].

**Fig. 7:**
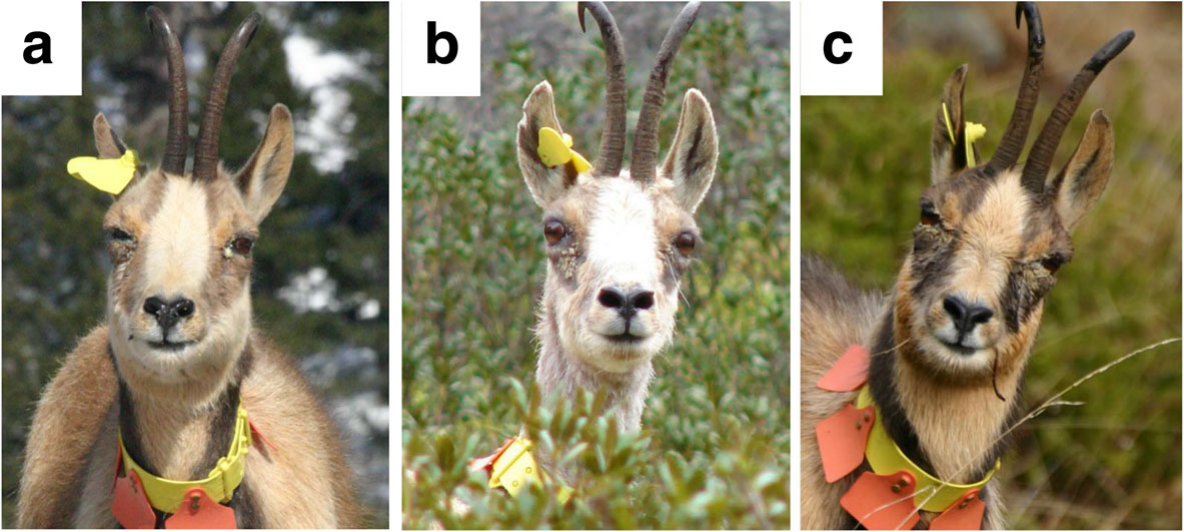
Male Pyrenean chamois affected twice by IKC. **a** 12 March 2007, bilateral excessive lachrymation with whitish deposits in the canthus medialis and partly closed eyelids, which appear swollen. **b** 1 June 2007, dry remnants of excessive lachrymation are visible on the fur under the eyes but the appearance of both eyes (including cornea and eyelids) is normal. **c** 4 December 2007, bilateral marked excessive lachrymation with new crust formation and swollen eyelids

The epidemic wave reached the sectors of Cauterets in the Pyrenees National Park in March 2007 (Fig. 2b–d). Chamois herds were successively infected and all individuals of a herd displayed IKC signs within a week. The outbreak was characterized by two peaks of morbidity (total case number and estimated prevalence) separated by 3 months, in spring 2007 and autumn/winter 2007–2008, respectively (Fig. 7), during which 63.5% of the recorded IKC-cases were observed.

The first peak (up to 76 cases within a month and 7.3% prevalence) corresponded to the disease spread within the chamois herds of the eastern part of the Gaube valley, and to cases in the south-western part of this valley (near the French-Spanish boundary). These cases in the south-west were the consequence of contacts with herds of the eastern side of the valley through the main mountain range of the boundary, which is the only known possibility of interactions between herds from the east and the west [34]. The cases in the south-west were observed in migrating herds coming from the north-west of the valley to graze in the south-west during summer (Fig. 2b–d).

The second peak reached up to 70 cases within a month and 23.3% prevalence, and was due to the disease spread in the north-western part of the valley. The first evidence of IKC in this zone corresponded to the return of the migrating chamois herds from their summer to their winter territory (Fig. 2b–d) [34].

**Fig. 8:**
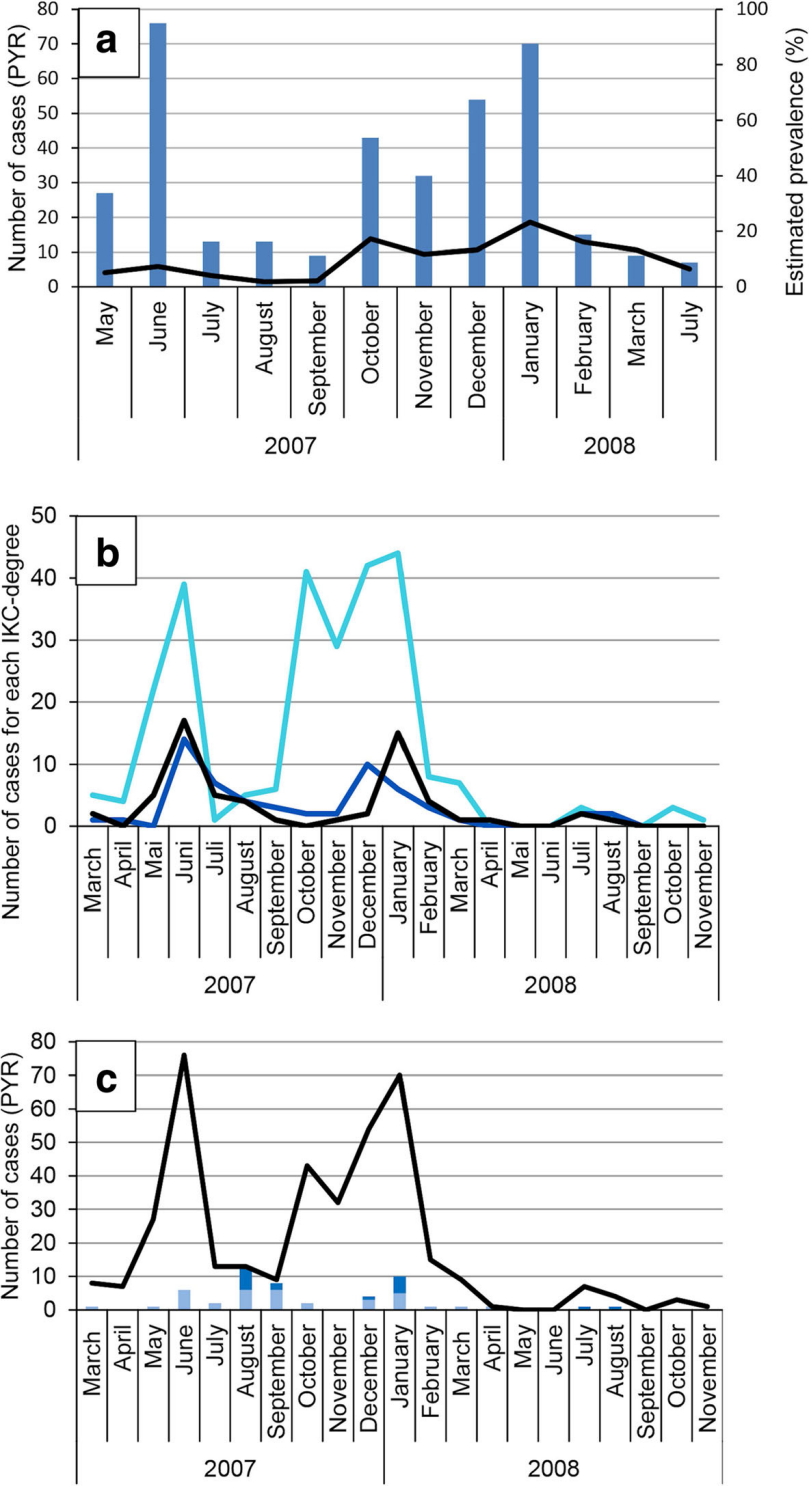
Number of IKC cases, prevalence, disease severity and mortality during the outbreak 2007–2008 in Cauterets. **a** Number of cases and prevalence. The number of disease cases *(blue bars*) and estimated prevalence (*black line*) are given for the two zones of Cauterets in the Pyrenees National Park, based on the field records from May 2007 to June 2008. **b** Disease severity. The number of disease cases (*solid lines*) is indicated according to the severity of the recorded ocular signs (classified according to Mavrot et al. [4]) and per month for the period from March 2007 to November 2008, as recorded in the zones of Cauterets in the Pyrenees National Park. *Light blue*: mild signs (ocular discharge only); *dark blue*: moderate signs (including corneal opacity); *black*: severe signs (including cornea neovascularization up to perforation). **c** Mortality. *The black solid line* indicates the total number of observed clinical cases. Bars represent the number of affected animals found dead*. Dark blue*: Males; *light blue*: Females

Severe ocular signs characterized by blindness, i.e. disease stages 2–3, were significantly more frequent during the first wave of the outbreak than during the second phase (Fig. 7; *P* = 0.0000), which was characterized by the highest prevalence (23.3%). The first wave extended from March to September 2007 and included 42.2% of all observed affected animals. The second wave extended from October 2007 to November 2008, with 23.5% of all observed affected animals. Furthermore, two marked adult chamois (a male and a female) were affected twice with IKC. The male, which was from the east, was affected for the first time during the first epidemic peak in the spring 2007, recovered from the disease and showed again IKC signs during the second epidemic peak in the fall 2007 (Fig. 8). The female, which was from the north-west, developed the disease for the first time in December 2007 and the second time in January 2008 after a transient absence of ocular signs. Additionally, another adult female developed IKC only 4 months after the other members of her group.

A total of 383 Pyrenean chamois were observed with IKC in Cauterets. The estimated prevalence was significantly higher in adults (10.5%, 95% CI 9.4–11.6) than in younger animals (4.2%, 95% CI 3.2–5.3; *P* = 0.0000). Prevalence did not differ between sexes among the diseased adults (*P* = 0.1417).

Mortality due to IKC was not recorded in kids and yearlings (except for two 1-year-old females) but in adults two peaks of mortality were observed, corresponding to the two peaks of morbidity: 29 carcasses were found in June-September 2007 (i.e. 55.8% of the total IKC-related adult mortality), and 14 carcasses (26.9%) were retrieved from December 2007 to January 2008 (Fig. 7c). The presence of orphans was recorded in 39 of 176 (22.2%) observed family groups, which was significantly more (*P* = 0.0000) than during the previous 13 years of observations in this region (71/2757, 2.6%) [J.-P. Crampe, unpubl. data].

Prevalence was significantly higher in the spring (calendar season and biological period; P ≤ 0.0011). It reached 20.4% (95% CI: 17.1–24.7) during the gestation period, dropped to 5.8% (4.8–7.0) during the birth period, remained stable during the lactation period (5.1%, 4.1–6.1) and increased again during the rut (12.7%, 10.3–15.4). The pattern for calendar seasons was similar.

Concerning the cluster analysis in Pyrenean chamois, three very similar strains were detected (Figs. 2d and 6, Additional file 3) in 36 animals, all with IKC signs. This cluster of strains was very different from the strains isolated in another region of the Spanish Pyrenees during the same period (Fig. 6) [8]. The first strain (PYR_07-08a; GenBank accession number KR052478) was detected in the whole area of Cauterets (Fig. 2d) in 27 animals and during the whole period of the outbreak. It was detected mostly in animals with severe ocular signs. The second strain (PYR_07b, GenBank accession number KR052472) differed in the *lppS* gene from the first by a deletion of 36 nucleotids (deletion of 12 amino acids) out of 767 and was only found in three animals, including two with severe IKC signs, from migrating herds sampled in the south-west of the Gaube valley (Fig. 2d). The third strain (PYR_07-08c, GenBank accession number KR052473) differed from the first by a deletion of 51 nucleotides (17 amino acids). It was identified in six animals in the north-west of the Gaube valley (Fig. 2d), which was the latest part of Cauterets affected by the outbreak. The appearence of this strain corresponded to the second peak of cases noticed in autumn/winter 2007–2008 (Fig. 6) but it was found mostly in animals with mild signs.

#### Southern French Alps

**Fig. 9:**
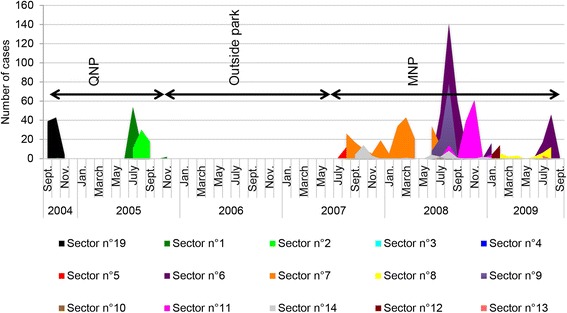
Number of IKC cases per sector during the 2004–2009 epidemic in the Southern French Alps. Each color corresponds to a different sector. Records were available only for the areas within the natural and national parks (Queyras (QNP) and Mercantour (MNP))

The outbreak started in 2003 and lasted more than 7 years (Figs. 3 and 9). It affected both Alpine chamois and Alpine ibex with comparable disease severity but IKC was observed in ibex as late as 4–6 months after the appearance of the first cases in chamois. Like in the Pyrenees, disease signs were often severe and orphans were often reported (detailed quantitative data not available). The outbreak began in Italy in the Valle Germanasca (Piemonte) in 2003. It spread to the south in France and Italy, following the main mountain range which represents the international border (details in Fig. 3b), and reached the south-western part of the Mercantour massif in 2009. This represents a 15 km spread per year for each year of the outbreak except for 2008 when 30 km were covered in the Mercantour massif. Prevalence and case number varied greatly among sectors and time periods (Fig. 9). Typically, a peak of cases was recorded when the disease reached a new sector and was followed by a prevalence decrease. However, a resurgence of cases was documented within three sectors (6, 7 and 9).

Like in the Pyrenees, the prevalence significantly differed between adults (12.7%, 95% CI 11.8–13.7) and younger chamois (9.5%, 95% CI 8.5–10.6; *P* = 0.0000), and there was no significant difference of prevalence between adult males and females (*P* = 0.1430) despite the higher percentage of females (62.1%) among the recorded cases (672 cases in all).

**Fig. 10:**
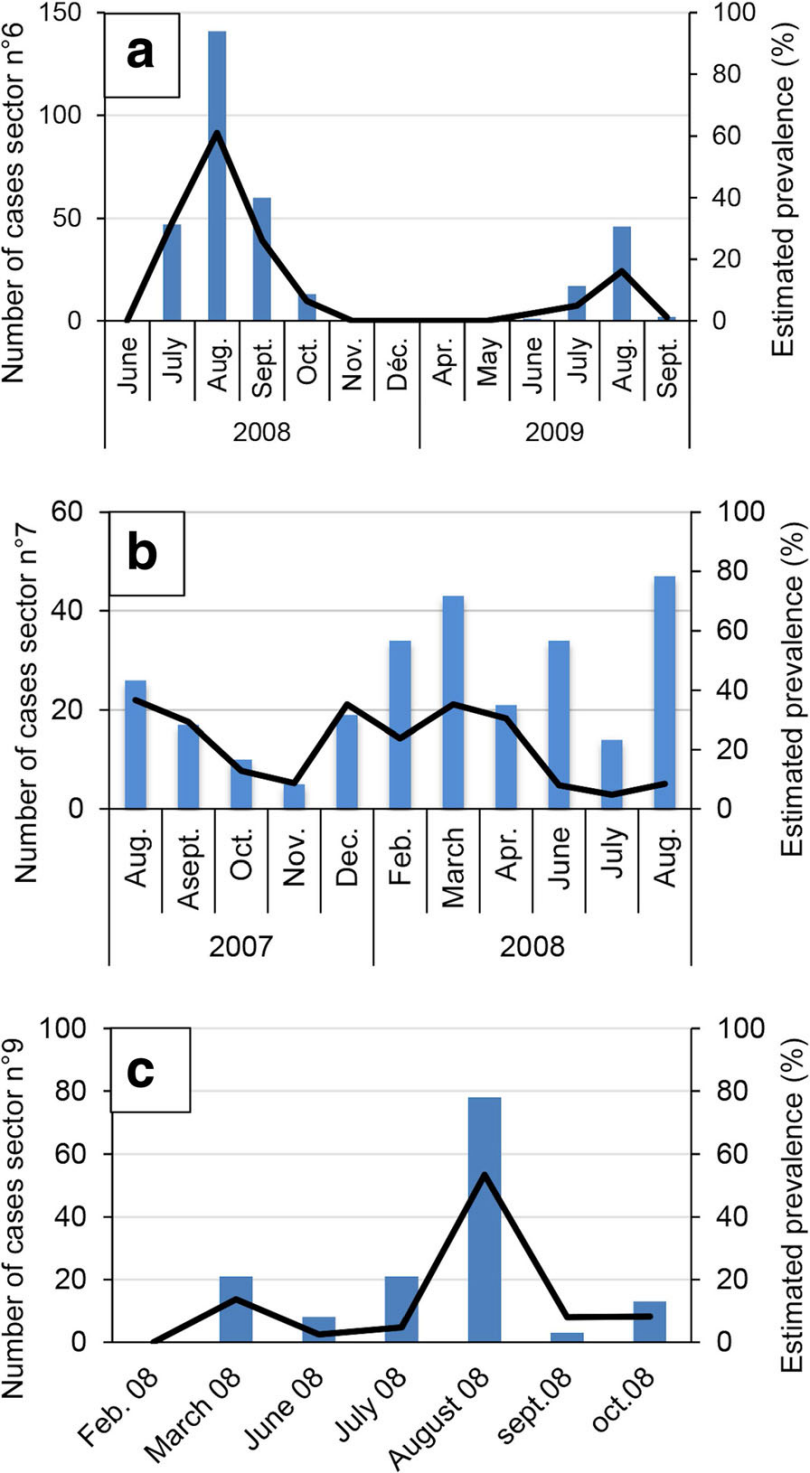
Number of IKC cases and disease prevalence in three sectors of the Southern French Alps. Information is based on the available field records from August 2007 to September 2009. *Blue bars* indicate the number of disease cases and *black lines* correspond to the estimated prevalence. **a** sector n°6, **b** sector n°7, **c** sector n°9

Two very similar strains were detected (Figs. 3d and 6) in 27 animals (both chamois and ibex), all symptomatic. The first strain (MER_04-09, GenBank accession number KR052477) was isolated from 2004 to 2009 (23 animals). It was present from Italy in the north to St-Etienne de Tinée in the south, corresponding to a distance of 75 km (Fig. 3d), and was identical to an Italian strain from the Val Thures in 2004 (strain 84) (Figs. 3d and 6). The second strain (MER_07-09, GenBank accession number KR052469) was different from the first by a deletion of 96 nucleotides (32 amino acids) out of 764. It was only found south of St. Etienne de Tinée and spread over 55 km from 2007 to 2009 (four animals) (Fig. 3d). In total, these two closely related strains covered a distance of 100 km from the location of the sequenced sample found farthest to the north to that collected in the most southern part of the study area, or a surface of approximately 17’000 km2 for the French part of the region affected by the outbreak. Like in Cauterets, the second peak in sector 7 (Figs. 4 and 10) coincided with the detection of a new strain (MER_07-09, two animals) that differed from the strain detected during the first peak (MER_04-09, one animal). Sequencing data were available only for the first peak in sector 6 (three animals, sequence MER_04-09) and for none of the affected animals in sector 9.

#### Ecrins

**Fig. 11:**
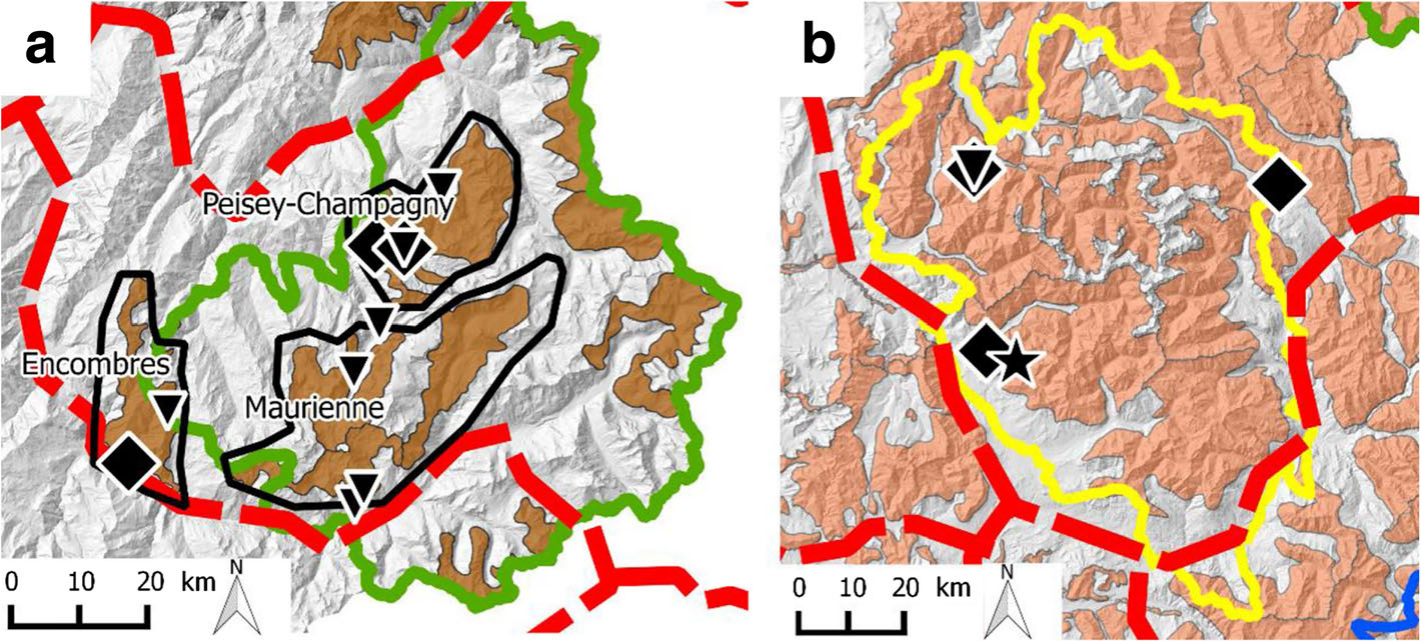
Detected *Mycoplasma conjunctivae* strains in the two study areas without severe outbreak. The *brown* areas indicate the distribution of ibex (**a**) or chamois (**b**). *Solid lines* correspond to the borders of the national parks. *Green*: Vanoise. *Yellow:* Ecrins. **a** Vanoise. The two identified strains (diamond and reverse triangle) confirm that the ibex population of Modane is in contact with the populations of Champagny and of Peisey-Champagny. **b** Ecrins. Three different strains (diamond, reverse triangle and star) of *M. conjunctivae* with one strain isolated twice in an interval of 5 years and a distance from each other of 45 km

Only sporadic cases of mild ocular disease in Alpine chamois were observed in the whole study area from 2004 to 2010, and both gamekeepers and veterinarians were uncertain as to whether this disease was really IKC. The cluster analysis revealed three *M. conjunctivae* strains from nine animals, which were very different from each other (Figs. 6 and 11). One strain (ECR_04-09, GenBank accession number KR052470) was found in three animals (sampled in 2002, 2005 and 2009, respectively). Two of them were from the same region, 45 km away from the location of the third animal (Fig. 11). The second strain (ECR_2005, GenBank accession number KR052471) was only found in one animal in 2005. The third strain (ECR_2010, GenBank accession number KR052474) was found in five animals (two asymptomatic) from a single region in 2010.

#### Vanoise

A weak epidemic wave was observed in 2006 in ibex, with associated mortality, but the local IKC situation is considered to be endemic with a few cases reported every year in chamois and ibex. The cluster analysis revealed two very different strains detected in ten ibex (Figs. 6 and 11). Both strains were found in symptomatic as well as in asymptomatic ibex, but the strain VAN_08 (GenBank accession number KR052475) was detected only in 2008 whereas the strain VAN_07-08 (GenBank accession number KR052476) was found both in 2007 and 2008. Furthermore, both strains were present in three different ibex colonies suspected to have contact with each other (Encombres, Maurienne, Peisey-Champagny; Fig. 11).

Overall, the Vanoise and Ecrins were characterized by an endemic situation, with a low number of cases and mild disease signs, and different clusters of strains. Furthermore, asymptomatic carriers were detected. By contrast, severe epidemics were observed in the Pyrenees and Southern French Alps, with a high morbidity, severe disease signs and associated mortality. In these two areas, few similar strains of *M. conjunctivae* were identified.

Comparison of the French strains identified with over 200 strains detected in former studies (database of the Institute of Veterinary Bacteriology, University of Bern) revealed that the French strains represented clusters that are different from those detected in the Swiss Alps and in the Spanish Pyrenees (Fig. 6).

## Discussion

Many IKC outbreaks have been observed in France since 1935 [32] but unlike in other European countries an etiological agent had not yet been identified in ibex and chamois. Here, we confirmed the etiological role of *M. conjunctivae* in IKC outbreaks affecting ibex and chamois populations both in the French Pyrenees and in the French Alps. Furthermore, we documented asymptomatic carriers in free-ranging wild Caprinae in France, corroborating observations in other European countries [4, 22, 30].

A number of ibex and chamois with typical severe IKC signs were tested negative, as previously observed [4, 22, 40]. It may be that secondary microbial agents compete with the mycoplasmas in late disease stages [5, 8, 22] or that part of the observed IKC-like signs are due to another etiology [4]. In addition, false negative results may be due to a reduced sensitivity of the test in highly contaminated samples.

We documented marked differences in the qPCR diagnostic success depending on both sample quality and the DNA extraction method. Based on our results, we recommend collecting eye swabs on the animals immediately rather than freezing heads and eyes before swab collection. Furthermore, we advise to directly extract DNA or at least conserve the samples in Guanidium buffer [41] rather than freezing the swabs and subsequently extracting the DNA [40].

### Disease course and host factors

According to our data, only ibex were affected during the mild outbreak of 2007 in the Vanoise, although chamois shared the same home ranges (ONCFS, Additional file 1). By contrast, during the Southern French Alps epidemics both ibex and chamois were observed to have typical ocular signs. It has been previously described that chamois which belong to a distinct taxonomic group (*Rupicapra* spp.) [47] are generally more often and more severely affected than ibex [4, 15, 29, 30], domestic goats (*Capra* spp.) and domestic sheep (*Ovis* spp.) [6], which are phylogenetically closely related to each other [47]. For the same degree of ocular signs, mycoplasma load in affected eyes has been shown to be higher in Alpine ibex than in Alpine chamois [4], claiming that chamois are particularly sensitive to *M. conjunctivae* infections. However, depending on the involved *M. conjunctivae* strain it also happens that only ibex are affected [4, 30] indicating some degree of strain-related host specificity.

In the Southern French Alps, both in this study and in former outbreaks [15, 29], ibex were affected at a later time point than chamois. Although there are exceptions, this suggests that IKC outbreaks tend to begin in chamois. Since ibex apparently need a higher mycoplasma load to develop disease signs [4], they may require more time to develop disease signs after an infection. Furthermore, when an epidemic starts in chamois, close interspecific interactions and a sufficient infection pressure are likely needed for ibex to become infected, which may contribute to the delay of the onset of the epidemics wave in this species.

Although mortality was high during epidemic outbreaks, our prevalence estimations put the impact of the disease on the population in perspective. We documented a significantly higher morbidity and higher mortality in adult animals compared to younger age classes in both sites with an epidemic pattern, in concordance with an unusually high occurrence of orphans. Although anecdotal, it is also interesting that the three asymptomatic carriers detected in the Ecrins were young animals. The lower susceptibility to IKC of the young age classes, characterized by a low morbidity and mortality and a higher proportion of healthy carriers, concurs with data from previous studies [4, 22] although a comparison was not always possible because either age classes were defined differently [48] or only the mortality was considered [2]. Based on similar observations in domestic sheep, Janovsky et al. [28] proposed that lambs play an important role in the persistence of *M. conjunctivae*. The weaker immune reactivity characterizing young individuals may reduce their susceptibility to mycoplasmoses because these are diseases resulting from adverse effects of the host’s immune reaction and inflammatory processes rather than from the direct toxicity of mycoplasma compounds [49–51].

Nevertheless, genetic differences among individuals may also influence susceptibility to infection and disease development, as already reported for a wide variety of mycoplasma diseases [52].

### Epidemic spread: role of social factors

In both study areas with an IKC epidemic (Pyrenees and Southern French Alps), prevalence was highest during the winter and spring rather than in summer, which contradicts former observations [6, 48]. This increased seasonal prevalence may be explained at least in part by the increase of animal movements and intraspecific contacts during the rut (from November to December [4]) and the resulting increased transmission of *M. conjunctivae* among social groups. The fact that in a former epidemic in Switzerland males were affected after females, with a peak of males affected during the rut [2], supports this suggestion. Furthermore, our data from the Pyrenees show the role of sexual segregation and movement of social groups in the pattern of spread of the disease. This was also observed in the Southern French Alps after the main IKC epidemic wave in summer 2005 in the Queyras, where spatial movements of affected young males during the rutting period contributed to disease spread (D. Gauthier, unpubl. obs.).

IKC spread twice as fast in the Mercantour National Park than in other parts of the Southern French Alps. As the estimated chamois density is higher in the Mercantour than in the rest of the Southern French Alps (Réseau Ongulés Sauvages, ONCFS/FNC/FDC, Additional file 1), population density may have additionally influenced IKC spread.

### Regional differences and strain virulence

We observed very different epidemiological scenarios depending on the study area. Beside host factors such as previous exposure to *M. conjunctivae,* environmental and population factors may contribute to regional differences [30, 31]. However, regions as different as the Pyrenees and the Southern Alps presented a similar disease pattern (severe epidemics), whereas the situation in the Southern French Alps was the opposite of that in both the Vanoise and Ecrins located close by (few mild cases).

The strains of *M. conjunctivae* detected in this study were site-specific: The same strains were identified within an epidemiological unit but strains differed among units. Our definition of an “epidemiological unit” was already supported by genetic data on Alpine ibex in Switzerland [53] and by telemetry studies in the Vanoise [46]. Here, our cluster analysis demonstrated that this definition is appropriate for the study of disease dynamics in mountain ungulates.

Our molecular data revealed the occurrence of few similar strains in areas with epidemics and of several very different strains in areas without epidemics, an observation already reported in the case of other mycoplasmoses in ruminants (*M. mycoides* subsp. *capri* [54] and *M. bovis* [55]) and of chytridiomycosis in amphibians (*Batrachochytrium dendrobatidis* [56]*)*. Mycoplasmas are characterized by a high diversity of surface antigens, generated by random combinatorial expression and high frequency variation of multiple membrane surface lipoproteins. These major coat proteins determine their ability to adhere to host cells and are the major targets of the host humoral immune response [57]. Since our cluster analysis was based on the lipoprotein S (LppS [42]), the different *M. conjunctivae* strains we detected may potentially differ in antigenicity and possibly also in virulence. Overall, these results suggest a role of pathogen factors (strain diversity and virulence) in the observed regional differences.

### Epidemic peaks and strain emergence

We documented that IKC can spread over mountain ranges and causes a peak of cases once it reaches a new sector, subsequently decreasing in prevalence or even vanishing at a local level. Considering the landscape and host population characteristics used to define a sector in this study, and the general stability of the detected strains in time and space, it is obvious that this outbreak dynamic was due to temporary pathogen “confinement” within a sector and subsequent pathogen introduction into naïve adjacent chamois groups via animal movements. However, we could show in the Pyrenees and in one Alpine sector that resurgence sometimes occurs within a sector and corresponds to the detection of new strains slightly different from the strain found during the first peak of cases. This suggests a clonal spread of *M. conjunctivae*, as was previously reported for other mycoplasmas such as *M. mycoides* subsp. *capri* in domestic goats [54] and *M. bovis* in cattle [55].

Successive epidemic waves have previously been reported in IKC outbreaks (e.g. [22, 29]) and two peaks were typically separated by 4 to 5 months. This time lapse may correspond to the time needed for the local population to recover from clinical signs and to clear the infection [3, 15, 30, 48]. The resurgence of IKC cases suggests that *M. conjunctivae* is able to escape host immunity. If the immune pressure gives rise to new strains, a recrudescence of cases may subsequently occur. In accordance with this hypothesis, in the Pyrenees the second IKC wave not only corresponded to the detection of a *M. conjunctivae* strain slightly different from the original one, it was also characterized by less severe disease signs than the initial case peak.

Furthermore, two marked individuals developed the disease a second time. The recovery period separating the clinical episodes in these chamois suggests that they underwent two consecutive infections. Trotter et al. [21] observed that antibodies produced against *M. conjunctivae* do not protect from re-infection. Similarly, vaccination attempts against mycoplasmoses did not protect from disease but even resulted in a worse disease course due to the artificially induced immune reaction [58–60]. By contrast, Baas et al. [1] documented that sheep infected twice showed milder ocular signs the second than the first time, and overall, the protective role of an acquired immunity against *M. conjunctivae* remains controversial. However, the virulence of the strain apparently influences the strength of the immune response, i.e., depending on the strain causing the first infection, the immune reaction and associated clinical outcome in case of a re-infection may greatly differ [54, 55, 61].

### Maintenance of *M. conjunctivae* in wild populations

Based on serological investigations, it had been previously proposed that *M. conjunctivae* does not persist in wildlife populations [6]. However, an IKC event may last from 2 to 9 years in wildlife [15]; *M. conjunctivae* is able to persist after recovery from clinical disease in both domestic and wild hosts [3, 21, 28] and the occurrence of healthy carriers in wild Caprinae is now established ([4, 22, 30], this study).

In agreement with a non-persistence of *M. conjunctivae* in the wild, domestic sheep have been proposed as a main infection source for wildlife [6, 26]. Every summer each of our study area hosts a mixture of sheep from various geographical origins. Considering that sheep are commonly infected with *M. conjunctivae* [6], we expected this diversity of sheep to be associated with numerous different *M. conjunctivae* strains. However, only two to three different strains were identified in each area, with a persistence of 3 to 6 years of the same strain independently of the epidemiological pattern. This is not in agreement with a yearly reintroduction of strains, and rather suggests that endemic *M. conjunctivae* strains circulate within wild ungulate populations. This hypothesis is also supported by the fact that IKC prevalence in wildlife is highest in the spring, i.e. before sheep occupy alpine pastures, and by the detection of infected asymptomatic ibex before the arrival of sheep [30].

Overall, there is increasing evidence in favor of a persistence of *M. conjunctivae* in the wild. Healthy carriers are often reported for mycoplasma species [54, 57, 62] and the ability of mycoplasmas to persist in a population is well-known and increasingly demonstrated by molecular evidence. For example, it had been assumed that new strains of *M. agalactiae* were recurrently introduced in the Pyrenees by imported sheep and responsible for the re-emergence of contagious agalactiae but it was recently demonstrated that a single cluster of *M. agalactiae* had been responsible for all known re-emergences and that it had persisted within the local sheep herds for 30 years [63].

Mycoplasmas are able to persist within a host by escaping the immune response of the host [50, 57, 62, 64] by various processes. Almost all of these processes induce changes in expression and/or structure of surface lipoproteins anchored in the mycoplasma membrane [57, 62, 64], and non-cytadhering mutants have been shown to be avirulent [65]. These mechanisms are crucial for the adaptation of mycoplasmas to their hosts and for the chronic colonization within a host [66]. In other words, immune pressure can induce antigenic modifications of surface proteins [67] and the immune system of the host represents an important selection factor for mycoplasmas [66]. Therefore, the severity of clinical signs in animals infected with *M. conjunctivae* is likely to result from both the ability of the host to develop an immune reaction towards a specific strain, and the ability of the strain to escape the immune response of a given host. Based on this knowledge, three scenarios have been proposed when a mycoplasma strain enters a host population able to develop an immune response [57, 62, 63, 68]: 1) the strain is efficiently combatted by the immune system of the hosts and vanishes from the population [33], likely after having caused severe disease signs in infected animals; 2) the strain undergoes mutations and an “equilibrium” between strain and host is achieved (mild signs, healthy carriers; [54, 55, 62] and particularities in Vanoise [15], Switzerland [4] and Ecrins [this study]); 3) the strain undergoes mutations and causes new epidemic waves until the situation evolves into scenario 1 or 2 (Pyrenees and Southern French Alps [this study]). In other words, a new outbreak could be due either to a mutation of a strain maintained in the population or to the introduction of new strains by other hosts such as domestic sheep. Nevertheless, our data suggest that this last scenario may be far less frequent than formerly assumed.

## Conclusion

With this study we confirmed the implication of *M. conjunctivae* in IKC in wild Caprinae in France, showed that the different outbreaks were not linked to each other, and demonstrated the persistence for at least 6 years of site-specific strains within local wild populations. Furthermore, we documented that strain characteristics, social interactions and landscape structure shape the dynamics of IKC outbreaks. Together with previous studies [4, 15, 31], this shows that IKC has a pronounced multifactorial character and that host, pathogen as well as environmental factors all play an important role in disease occurrence, spread and severity. Importantly, IKC presents features inherent in the biology of other mycoplasmas. From a technical point of view, this work demonstrates the usefulness of performing long-term studies, joining field observations and molecular analyses, and harmonizing data collection in different study sites to track factors influencing the dynamic of infectious disease outbreaks in free-ranging wildlife [69]. It also illustrates the necessity to define epidemiological units to document and possibly predict the spread of infectious agents transmitted mainly by direct contact. IKC, which is a visible disease, may well represent an interesting model to study the epidemiology of diseases with similar transmission routes in wildlife populations.

## Additional files

Additional file 1: Spatial distribution and estimated densities of wild mountain ungulates in the two study regions in 2010. Densities are expressed in animals/km^2^ and base on direct animal counts. A: French Alps; B: French Pyrenees (source: ONCFS [44]). (TIF 23772 kb)
Additional file 2: French departments from which the domestic sheep grazing in the different national and regional parks of the study areas come. The departments sending sheep to the Pyrenees (red solid line) for the summer grazing season are colored in pale red, to the Vanoise (green solid line) are in pale green, to the Ecrins (yellow solid line) are in pale yellow, to the Queyras Regional Natural Park (dark blue solid line) are in pale purple, and to the Mercantour (royal blue solid line) are in pale blue. Pies indicate the percentage of sheep living in the park all year round (light blue) compared to those coming from other departments (dark blue). (TIF 4821 kb)
Additional file 3: List of the detected strains. The accession number in GenBank, the animals from which the strains come from, and details about samples, signs of infectious keratoconjunctivitis (IKC), date of sampling and geographic origin of the animals are indicated for each strain. (DOCX 17 kb)
