# Supplementary figures and images for: Infectious keratoconjunctivitis in wild Caprinae: merging field observations and molecular analyses sheds light on factors shaping outbreak dynamics
Source: BMC Vet Res. 2017 Mar 4;13:67. doi: 10.1186/s12917-017-0972-0 (PMC5336646; doi:10.1186/s12917-017-0972-0)

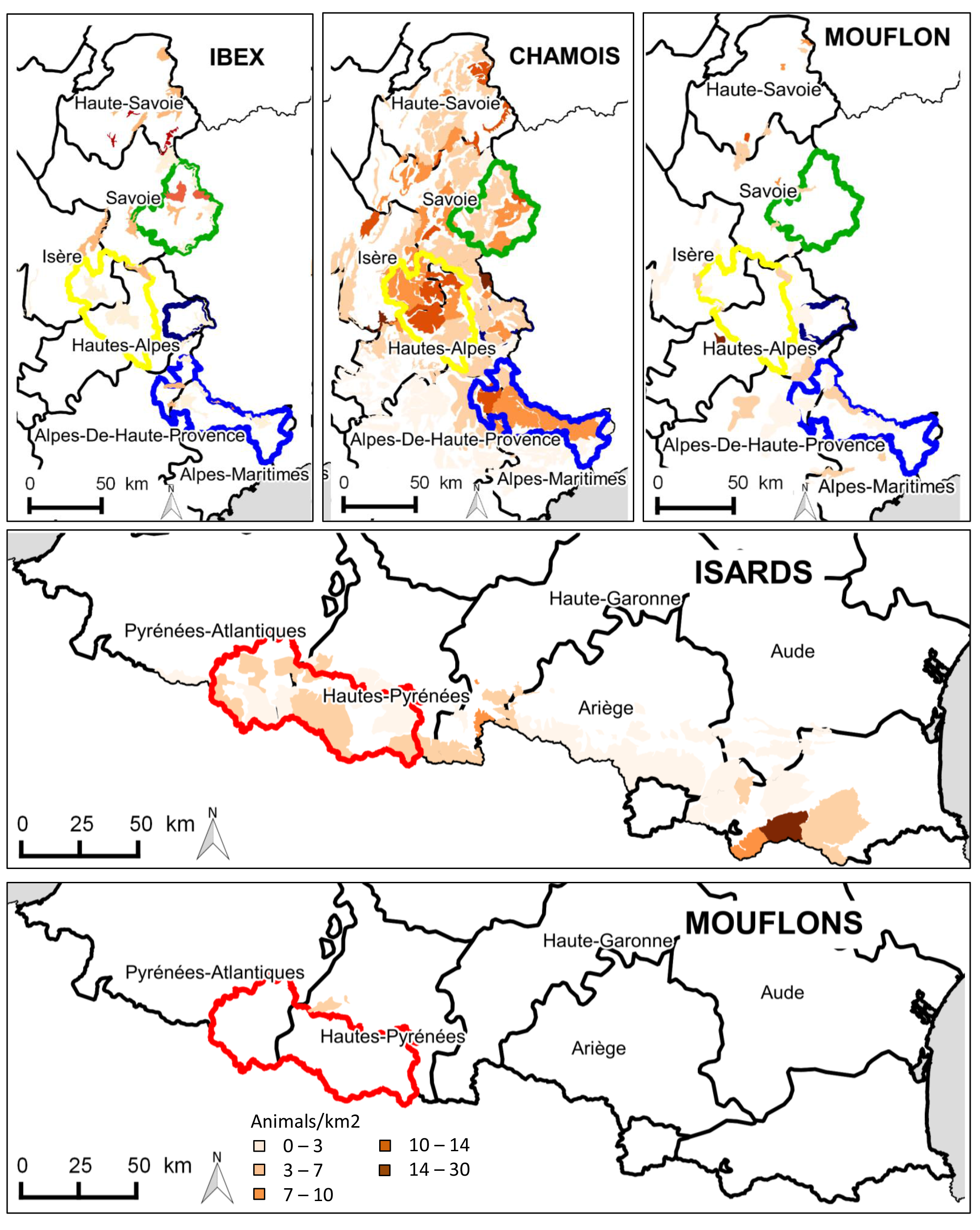

Supplement: Additional file 1: — Spatial distribution and estimated densities of wild mountain ungulates in the two study regions in 2010. Densities are expressed in animals/km2 and base on direct animal counts. A: French Alps; B: French Pyrenees (source: ONCFS [44]). (TIF 23772 kb) [file 12917_2017_972_MOESM1_ESM.tif]

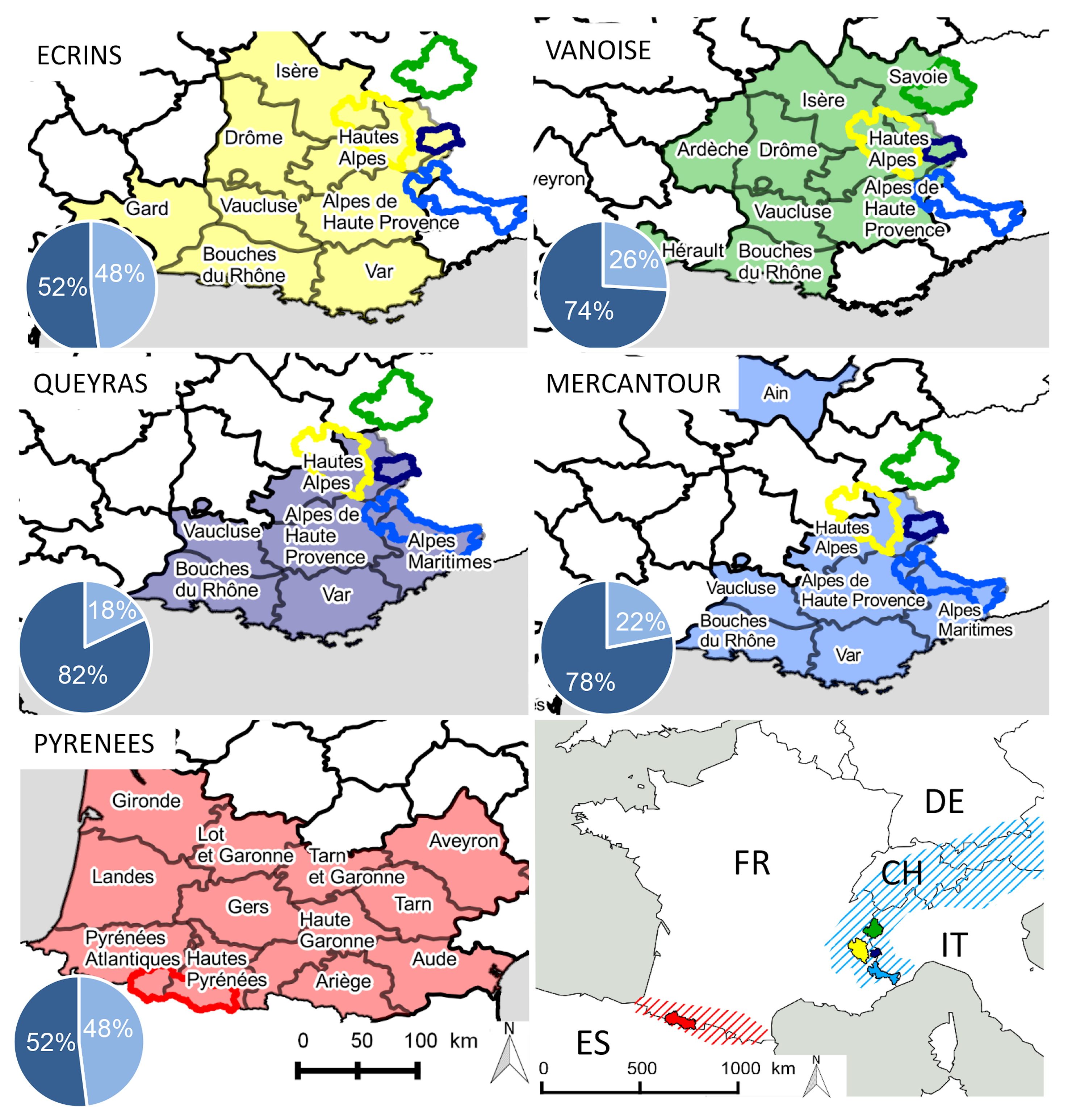

Supplement: Additional file 2: — French departments from which the domestic sheep grazing in the different national and regional parks of the study areas come. The departments sending sheep to the Pyrenees (red solid line) for the summer grazing season are colored in pale red, to the Vanoise (green solid line) are in pale green, to the Ecrins (yellow solid line) are in pale yellow, to the Queyras Regional Natural Park (dark blue solid line) are in pale purple, and to the Mercantour (royal blue solid line) are in pale blue. Pies indicate the percentage of sheep living in the park all year round (light blue) compared to those coming from other departments (dark blue). (TIF 4821 kb) [file 12917_2017_972_MOESM2_ESM.tif]
