# Supplementary material for: Infectious keratoconjunctivitis in wild Caprinae: merging field observations and molecular analyses sheds light on factors shaping outbreak dynamics
Source: BMC Vet Res. 2017 Mar 4;13:67. doi: 10.1186/s12917-017-0972-0 (PMC5336646; doi:10.1186/s12917-017-0972-0)
Supplement: Additional file 3: — List of the detected strains. The accession number in GenBank, the animals from which the strains come from, and details about samples, signs of infectious keratoconjunctivitis (IKC), date of sampling and geographic origin of the animals are indicated for each strain. (DOCX 17 kb) [file 12917_2017_972_MOESM3_ESM.docx]

| **Sample Nr.** | **Accession numbers** | **Animal from which sample was taken** | **Organ from which sample was taken** | **symptoms of individual** | **year** | **place** | **Numbers of animals presenting the considered strain** |
| --- | --- | --- | --- | --- | --- | --- | --- |
| PYR_07-08a | KR052478 | Pyrenean Chamois, male, 7 years old | Frozen head | Left eye with ulceration of the cornea, presence of ocular lacrymation | Aug. 2007 | Pyrenees, France | 27 Pyrenean Chamois, all with ocular (IKC) signs |
| PYR_07b | KR052472 | Pyrenean Chamois, female, 13 years old | Frozen head | Both eyes with perforation of the cornea, presence of ocular lacrymation | Sept. 2007 | Pyrenees, France | 3 Pyrenean Chamois |
| PYR_07-08c | KR052473 | Pyrenean Chamois, Female, 7 years old | Frozen eyes | Opacification of both eyes’ cornea, presence of ocular lacrymation | Feb. 2008 | Pyrenees, France | 6 Pyrenean Chamois |
| MER_04-09 | KR052477 | Alpine Chamois, female, 4 years old | Frozen eyes | Both eyes with perforation of the cornea, presence of ocular lacrymation | Feb. 2004 | Southern French Alps, France | 20 Alpine Chamois, 2 ibex |
| MER_07-09 | KR052469 | Alpine Chamois, female, 9 years old | Frozen head | Opacification of both eyes’ cornea, presence of ocular lacrymation | Jan. 2009 | Southern French Alps, France | 4 Alpine Chamois |
| ECR_04-09 | KR052470 | Alpine Chamois, male, 1 year old | Frozen eyes | Presence of ocular lacrymation | Oct. 2009 | Ecrins National Park, France | 3 Alpine Chamois |
| ECR_2005 | KR052471 | Alpine Chamois, female, 1 year old | Frozen eyes | Opacification of both eyes’ cornea, presence of ocular lacrymation | Sept. 2005 | Ecrins National Park, France | 1 Alpine Chamois |
| ECR_2010 | KR052474 | Alpine Chamois, male, kid | Frozen eye swabs | No ocular signs | Nov. 2010 | Ecrins National Park, France | 5 Alpine Chamois |
| VAN_07-08 | KR052476 | Ibex, female 8 years old | Frozen eyes | Both eyes with perforation of the cornea, presence of ocular lacrymation | July 2008 | Vanoise National Park, France | 7 Ibex |
| VAN_08 | KR052475 | Ibex, male, 7 years old | Frozen eye swabs | No ocular signs | Apr. 2008 | Vanoise National Park, France | 3 Ibex |
